# Prospective mapping of viral mutations that escape antibodies used to treat COVID-19

**DOI:** 10.1126/science.abf9302

**Published:** 2021-01-25

**Authors:** Tyler N. Starr, Allison J. Greaney, Amin Addetia, William W. Hannon, Manish C. Choudhary, Adam S. Dingens, Jonathan Z. Li, Jesse D. Bloom

**Affiliations:** 1 Basic Sciences and Computational Biology, Fred Hutchinson Cancer Research Center, Seattle, WA 98109, USA.; 2 Department of Genome Sciences, University of Washington, Seattle, WA 98109, USA.; 3 Medical Scientist Training Program, University of Washington, Seattle, WA 98109, USA.; 4 Molecular and Cellular Biology Graduate Program, University of Washington, Seattle, WA 98109, USA.; 5 Brigham and Women’s Hospital, Harvard Medical School, Boston, MA 02115, USA.; 6 Howard Hughes Medical Institute, Seattle, WA 98109, USA.

## Abstract

Antibodies are a potential therapy for severe acute respiratory syndrome coronavirus 2 (SARS-CoV-2), but the risk of the virus evolving to escape them remains unclear. Here we map how all mutations to the receptor binding domain (RBD) of SARS-CoV-2 affect binding by the antibodies in the REGN-COV2 cocktail and the antibody LY-CoV016. These complete maps uncover a single amino acid mutation that fully escapes the REGN-COV2 cocktail, which consists of two antibodies, REGN10933 and REGN10987, targeting distinct structural epitopes. The maps also identify viral mutations that are selected in a persistently infected patient treated with REGN-COV2 and during in vitro viral escape selections. Finally, the maps reveal that mutations escaping the individual antibodies are already present in circulating SARS-CoV-2 strains. These complete escape maps enable interpretation of the consequences of mutations observed during viral surveillance.

## Mapping antibody escape in SARS-CoV-2

Several antibodies are in use or under development as therapies to treat COVID-19. As new severe acute respiratory syndrome coronavirus 2 (SARS-CoV-2) variants emerge, it is important to predict whether they will remain susceptible to antibody treatment. Starr *et al.* used a yeast library that covers all mutations to the SARS-CoV-2 receptor-binding domain that do not strongly disrupt binding to the host receptor (ACE2) and mapped how these mutations affect binding to three leading anti–SARS-CoV-2 antibodies. The maps identify mutations that escape antibody binding, including a single mutation that escapes both antibodies in the Regeneron antibody cocktail. Many of the mutations that escape single antibodies are circulating in the human population.

*Science*, this issue p. 850

## Teaser

Complete maps of SARS-CoV-2 mutations that escape the Regeneron monoclonal antibody cocktail help explain viral evolution in a treated patient.

Antibodies are being developed as therapeutics to combat severe acute respiratory syndrome coronavirus 2 (SARS-CoV-2) (*1*). Antibodies against some other viruses can be rendered ineffective by viral mutations that are selected during treatment of infected patients (*2*, *3*) or that spread globally to confer resistance on entire viral clades (*4*). Therefore, determining which SARS-CoV-2 mutations escape key antibodies is essential for assessing how mutations observed during viral surveillance may affect the efficacy of antibody treatments.

Most leading anti–SARS-CoV-2 antibodies target the viral receptor binding domain (RBD), which mediates binding to the angiotensin-converting enzyme 2 (ACE2) receptor (*5*, *6*). We recently developed a deep mutational scanning method to map how all mutations to the RBD affect its function and recognition by antiviral antibodies (*7*, *8*). This method involves creating libraries of RBD mutants, expressing them on the surface of yeast, and using fluorescence-activated cell sorting and deep sequencing to quantify how each mutation affects RBD folding, ACE2 affinity (measured across a titration series), and antibody binding (fig. S1A). In this study, we used the duplicate mutant libraries described in (*7*), which consist of barcoded RBD variants that cover 3804 of the 3819 possible amino acid mutations. Our libraries were made in the genetic background of the RBD from the early isolate Wuhan-Hu-1, which still represents the most common RBD sequence, although several mutants are currently increasing in frequency (*9*, *10*). We mapped how the 2034 mutations that do not strongly disrupt RBD folding and ACE binding (*7*) affected binding by recombinant forms of the two antibodies in Regeneron’s REGN-COV2 cocktail (REGN10933 and REGN10987) (*11*, *12*) and Eli Lilly’s LY-CoV016 antibody (also known as CB6 or JS016) (*13*) (fig. S1B). REGN-COV2 was recently granted emergency use authorization for treatment of COVID-19 (*14*), whereas LY-CoV016 is currently in phase 3 clinical trials (*15*).

**Fig. 1:**
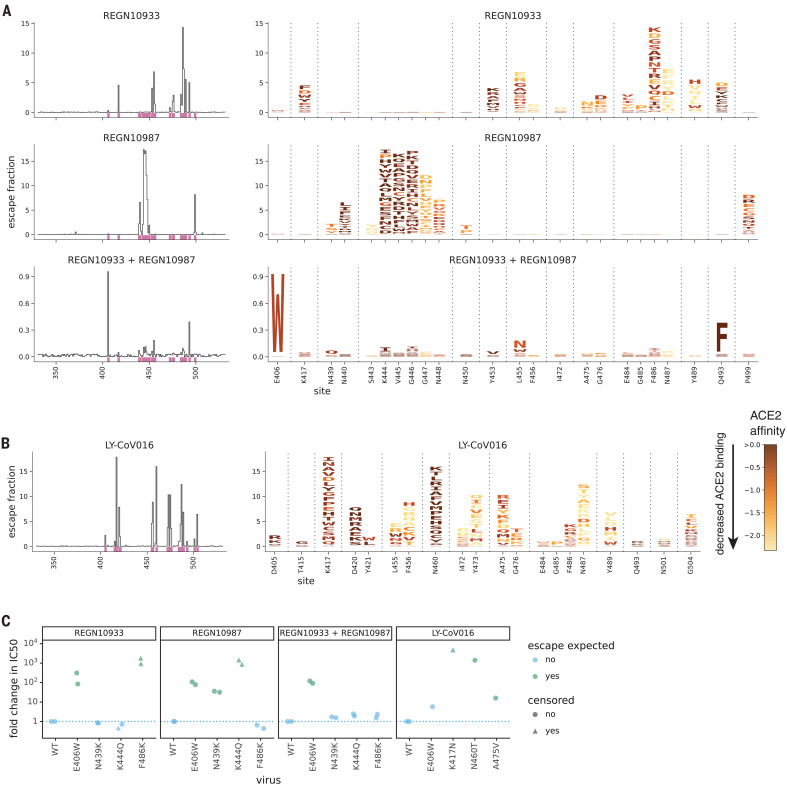
Complete maps of mutations that escape binding by the REGN-COV2 antibodies and Ly-CoV016. (**A**) Maps for antibodies in REGN-COV2. Line plots at left show escape at each site in the RBD (summed effects of all mutations at each site). Sites of strong escape (purple underlines) are shown in logo plots at right. The height of each letter is proportional to how strongly that amino acid mutation mediates escape, with a per-mutation “escape fraction” of 1 corresponding to complete escape. The *y*-axis scale is different for each row, so, for instance, E406W escapes all REGN antibodies but is most visible for the cocktail as it is swamped out by other sites of escape for the individual antibodies. See https://jbloomlab.github.io/SARS-CoV-2-RBD_MAP_clinical_Abs/ for zoomable versions. Letters are colored according to how mutations affect the RBD’s affinity for ACE2 as measured via yeast display (*7*), with yellow indicating poor affinity and brown indicating good affinity; see fig. S2, A and B, for maps colored by how mutations affect expression of folded RBD and fig. S2, C and D, for distribution of effects on ACE2 affinity and RBD expression across all mutations observed among circulating viral isolates. (**B**) Map, as in (A), for LY-CoV016. (**C**) Validation of key mutations in neutralization assays using spike-pseudotyped lentiviral particles. We chose to validate mutations predicted to have large effects or that are present at high frequency among circulating SARS-CoV-2 isolates (e.g., N439K). Each point indicates the fold increase in the median inhibitory concentration (IC_50_) for a mutation relative to the unmutated wild-type (WT) spike, which contains D614G. The dotted blue line at 1 indicates WT-like neutralization, and values >1 indicate increased neutralization resistance. Point colors indicate whether escape was expected at that site from the maps. Point shapes indicate that the fold change is censored (an upper or lower limit) owing to the IC_50_ being outside the dilution series used. Most mutants were tested in duplicate and thus have two points. Full neutralization curves are shown in fig. S3. Single-letter abbreviations for the amino acid residues are as follows: A, Ala; C, Cys; D, Asp; E, Glu; F, Phe; G, Gly; H, His; I, Ile; K, Lys; L, Leu; M, Met; N, Asn; P, Pro; Q, Gln; R, Arg; S, Ser; T, Thr; V, Val; W, Trp; and Y, Tyr.

We completely mapped RBD mutations that escape binding by the three individual antibodies as well as the REGN-COV2 cocktail (Fig. 1, A and B, and zoomable maps at https://jbloomlab.github.io/SARS-CoV-2-RBD_MAP_clinical_Abs/). REGN10933 and REGN10987 are escaped by largely nonoverlapping sets of mutations in the RBD’s receptor binding motif (Fig. 1A), consistent with structural work showing that these antibodies target distinct epitopes in this motif (*11*). But unexpectedly, one mutation [Glu^406^→Trp (E406W)] strongly escapes the cocktail of both antibodies (Fig. 1A). The escape map for LY-CoV016 also reveals escape mutations at a number of different sites in the RBD (Fig. 1B). Although some escape mutations impair the RBD’s ability to bind ACE2 or be expressed in properly folded form, many come at little or no cost to these functional properties, according to prior deep mutational scanning measurements using yeast-displayed RBD (*7*) (color gradient in Fig. 1, A and B, indicates loss of ACE2 affinity and in fig. S2 indicates decrease in RBD expression).

To validate the antigenic effects of key mutations, we performed neutralization assays using spike-pseudotyped lentiviral particles and found concordance between the antibody binding escape maps and neutralization assays (Fig. 1C and fig. S3). As expected from the maps for the REGN-COV2 antibodies, a mutation at site 486 escaped neutralization only by REGN10933, whereas mutations at sites 439 and 444 escaped neutralization only by REGN10987—and so none of these mutations escaped the cocktail. But E406W escaped both individual REGN-COV2 antibodies and thus also strongly escaped the cocktail. Structural analyses and viral-escape selections led Regeneron to posit that no single amino acid mutation could escape both antibodies in the cocktail (*11*, *12*), but our complete maps identify E406W as a cocktail escape mutation. E406W affects the REGN-COV2 antibodies in a relatively specific way and does not grossly perturb the function of the RBD, given that it only mildly reduces neutralization by LY-CoV016 (Fig. 1C) and the titers of spike-pseudotyped lentiviral particles (fig. S3F).

**Fig. 2:**
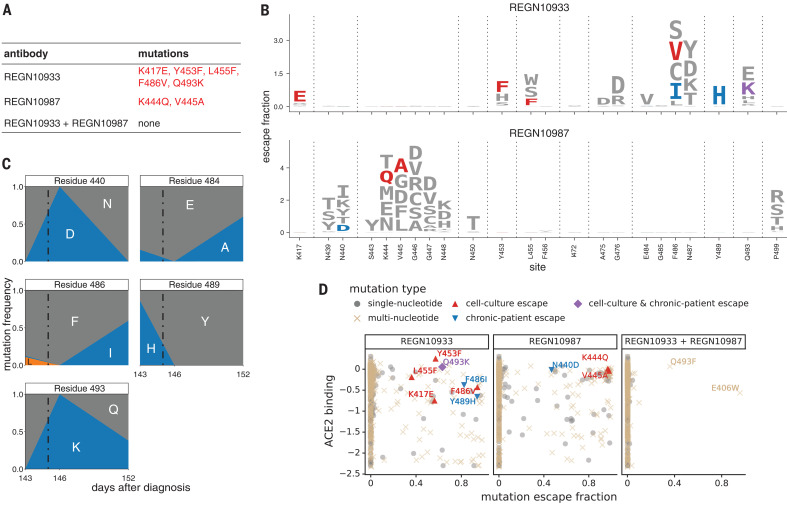
Escape maps are consistent with viral mutations selected in cell culture and a persistently infected patient. (**A**) Viral escape mutations selected by Regeneron with spike-pseudotyped VSV in cell culture in the presence of antibody (*12*). (**B**) Escape maps like those in Fig. 1A, but showing only mutations accessible by single-nucleotide changes to the Wuhan-Hu-1 sequence, with nongray colors indicating mutations in cell culture (red), the infected patient (blue), or both (purple). Figure S5 shows these maps colored by how mutations affect ACE2 affinity or RBD expression. (**C**) Dynamics of RBD mutations in a patient treated with REGN-COV2 at day 145 of infection (black dot-dash vertical line). E484A rose in frequency in linkage with F486I, but because E484A is not an escape mutation in our maps, it is not shown in other panels. See also fig. S4. (**D**) The escape mutations that arise in cell culture and the infected patient are single-nucleotide–accessible and escape antibody binding without imposing a large cost on ACE2 affinity [as measured using yeast display (*7*)]. Each point is a mutation, with shape and color indicating whether it is accessible and selected during viral growth. Points farther to the right on the *x* axis indicate stronger escape from antibody binding; points higher up on the *y* axis indicate higher ACE2 affinity.

To explore whether our escape maps are consistent with how the virus evolves under antibody selection, we first examined data from Regeneron’s viral escape–selection experiments in which spike-expressing vesicular stomatitis virus (VSV) was grown in cell culture in the presence of either REGN10933, REGN10987, or the REGN-COV2 cocktail (*12*). That work identified five escape mutations from REGN10933, two from REGN10987, and none from the cocktail (Fig. 2A). All seven cell culture–selected mutations were prominent in our escape maps while also being accessible by just a single-nucleotide change to the wild-type codon in the Wuhan-Hu-1 RBD sequence (Fig. 2B), demonstrating concordance between the escape maps and viral evolution under antibody pressure in cell culture. Notably, E406W is not accessible by a single-nucleotide change, which may explain why it was not identified by the Regeneron cocktail selections despite being relatively well tolerated for RBD folding and ACE2 affinity.

To determine whether the escape maps could inform analysis of viral evolution in infected humans, we examined deep sequencing data from a persistently infected immunocompromised patient who was treated with REGN-COV2 at day 145 after diagnosis with COVID-19 (*16*). The late administration of the treatment allowed ample time for the patient’s viral population to accumulate genetic diversity, some of which could have been driven by immune pressure, because the patient mounted a weak autologous neutralizing antibody response before treatment (*16*). Administration of REGN-COV2 was followed by rapid changes in the frequencies of five amino acid mutations in the RBD (Fig. 2C and fig. S4). Our escape maps showed that three of these mutations escaped REGN10933 and that one escaped REGN10987 (Fig. 2B). Notably, the mutations did not all sweep to fixation after antibody treatment; instead, there were competing rises and falls (Fig. 2C). This pattern has been observed in the adaptive within-host evolution of other viruses (*17*, *18*) and can arise from genetic hitchhiking and competition among viral lineages. Both these forces appear to be at play in the persistently infected patient (Fig. 2C and fig. S4C): E484A (not an escape mutation in our maps) hitchhikes with F486I (which escapes REGN10933) after treatment, and the viral lineage carrying N440D and Q493K (which escape REGN10987 and REGN10933, respectively) competes first with the REGN10933 escape-mutant Y489H and then with the lineage carrying E484A and F486I and the Q493K lineage.

Three of the four escape mutations in the REGN-COV2–treated patient were not identified in Regeneron’s viral cell culture selections (Fig. 2B), illustrating an advantage of complete maps. Viral selections are incomplete in the sense that they only identify whatever mutations are stochastically selected in that particular cell culture experiment. In contrast, complete maps annotate all mutations, which could include mutations that arise for reasons unrelated to treatment but incidentally affect antibody binding.

Of course, viral evolution is shaped by functional constraints as well as pressure to evade antibodies. The mutations selected in cell culture and the patient consistently met the following criteria: They escaped antibody binding, were accessible via a single-nucleotide change, and imposed little or no cost on ACE2 affinity [as measured by prior deep mutational scanning using yeast-displayed RBD (*7*)] (Fig. 2D and fig. S5). Therefore, complete maps of how mutations affect key biochemical phenotypes of the RBD (e.g., ACE and antibody binding) can be used to assess likely paths of viral evolution. One caveat is that over longer evolutionary time frames, the space of tolerated mutations could shift as a result of epistatic interactions, as has been observed in viral immune and drug escape (*19*–*21*).

**Fig. 3:**
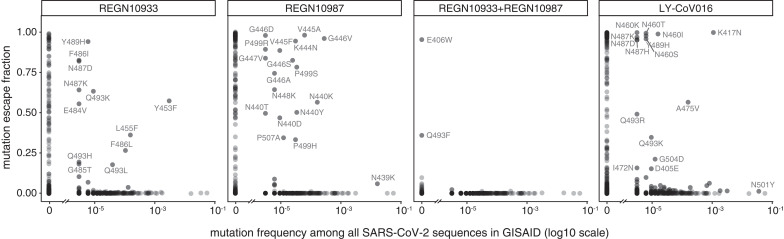
Antibody escape mutations in circulating SARS-CoV-2. For each antibody or antibody combination, the escape score for each mutation is plotted versus its frequency among the 317,866 high-quality human-derived SARS-CoV-2 sequences on GISAID (*26*) as of 11 January 2021. Escape mutations with notable GISAID frequencies are labeled. The REGN-COV2 cocktail escape mutation E406W requires multiple nucleotide changes from the Wuhan-Hu-1 RBD sequence and is not observed among sequences in GISAID. Other mutations to residue E406 (E406Q and E406D) are observed with low frequency counts, but neither of these mutant amino acids is a single-nucleotide mutation away from W either.

The complete maps enable us to assess what escape mutations are already present among circulating SARS-CoV-2. We examined all human-derived SARS-CoV-2 sequences available as of 11 January 2021 and found a substantial number of RBD mutations that escaped one or more of the antibodies (Fig. 3). However, the only escape mutations present in >0.1% of sequences were the REGN10933 escape-mutant Y453F [0.3% of sequences; see also (*12*)], the REGN10987 escape-mutant N439K [1.7% of sequences; see also Fig. 1C and (*22*)], and the LY-CoV016 escape-mutant K417N (0.1% of sequences; see also Fig. 1C). Y453F is associated with independent outbreaks linked to mink farms in the Netherlands and Denmark (*23*, *24*); notably, the mink sequences themselves sometimes also contain other escape mutations, such as F486L (*24*). N439K is prevalent in Europe, where it has constituted a large percentage of sequences from regions including Scotland and Ireland (*22*, *25*). K417N is present in the B.1.351 lineage first identified in South Africa (*10*). Another mutation of current interest is N501Y, which is present in B.1.351 and also the B.1.1.7 lineage originally identified in the United Kingdom (*9*). Our maps indicate that N501Y has no effect on either of the REGN-COV2 antibodies and has only a modest effect on LY-CoV016 (Fig. 3).

**Fig. 4:**
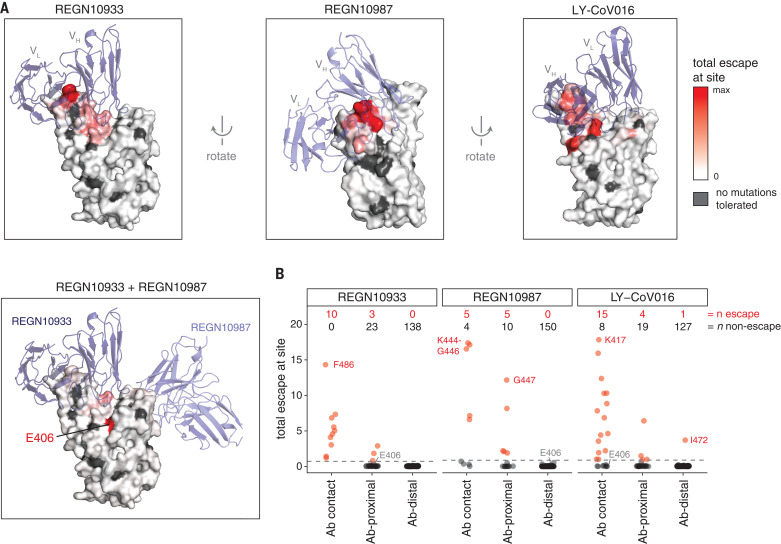
Structural context of escape mutations. (**A**) Escape maps projected on antibody-bound RBD structures. [REGN10933 and REGN10987: Protein Data Bank (PDB) ID 6XDG (*11*); LY-CoV016: PDB ID 7C01 (*13*)]. Antibody heavy- and light-chain variable domains are shown as blue cartoons, and the RBD surface is colored to indicate how strongly mutations at that site mediate escape (white indicates no escape, red indicates strongest escape site for that antibody or cocktail). Sites where no mutations are functionally tolerated are colored gray. (**B**) For each antibody, sites were classified as direct antibody contacts (non-hydrogen atoms within 4 Å of antibody), antibody-proximal (4 to 8 Å), or antibody-distal (>8 Å). Each point indicates a site, classified as escape (red) or non-escape (black). The dashed gray line indicates the cutoff used to classify sites as escape or non-escape (see materials and methods for details). Red and black numbers indicate how many sites in each category are escape or non-escape sites, respectively. Interactive visualizations are at https://jbloomlab.github.io/SARS-CoV-2-RBD_MAP_clinical_Abs/, and hypothesized mechanisms of escape and additional structural details for labeled points are shown in fig. S6.

To determine whether the escape maps could be rationalized from the structural interfaces of the antibodies and RBD, we projected the maps onto crystal or cryo–electron microscopy structures (Fig. 4A; interactive versions at https://jbloomlab.github.io/SARS-CoV-2-RBD_MAP_clinical_Abs/). As might be expected, escape mutations generally occur in the antibody-RBD interface. However, structures alone are insufficient to predict which mutations mediate escape. For example, LY-CoV016 uses both its heavy and light chains to bind a wide epitope overlapping the ACE2-binding surface, but escape is dominated by mutations at RBD residues that contact the heavy-chain complementarity-determining regions (Fig. 4A and fig. S6, E to G). In contrast, escape from REGN10933 and REGN10987 mostly occurs at RBD residues that pack at the antibody heavy- and light-chain interface (Fig. 4A and fig. S6, A to D). The E406W mutation that escapes the REGN-COV2 cocktail occurs at a residue not in contact with either antibody (Fig. 4, A and B). Although E406 is in closer structural proximity to LY-CoV016 (Fig. 4B and fig. S6H), the E406W mutation has a much smaller impact on this antibody (Fig. 1, B and C), suggesting a long-range structural mechanism specific to the REGN-COV2 antibodies (fig. S6I). Taken together, mutations at RBD residues that contact antibody do not always mediate escape, and several prominent escape mutations occur at residues not in contact with antibody (Fig. 4B and fig. S6, D and G).

In this study, we have completely mapped mutations that escape three leading anti–SARS-CoV-2 antibodies. These maps demonstrate that prior characterization of escape mutations was incomplete, having identified neither a single amino acid mutation that escapes both antibodies in the REGN-COV2 cocktail nor most mutations that arose in a persistently infected patient treated with the cocktail. Of course, our maps still do not answer the most pressing question: Will SARS-CoV-2 evolve widespread resistance to these antibodies? But certainly, it is concerning that so many escape mutations impose little cost on RBD folding or receptor affinity and that some are already present at low levels among circulating viruses. Ultimately, it will be necessary to wait and see what mutations spread as SARS-CoV-2 circulates in the human population. Our work will help with the “seeing,” by enabling immediate interpretation of the effects of the mutations cataloged by viral genomic surveillance.

## Supplementary Materials

science.sciencemag.org/content/371/6531/850/suppl/DC1 Materials and Methods Figs. S1 to S6 References (*29*–*39*) MDAR Reproducibility Checklist Data S1

## Appendix

View/request a protocol for this paper from *Bio-protocol*.
